# Coronavirus disease 2019 among pregnant Chinese women: case series data on the safety of vaginal birth and breastfeeding

**DOI:** 10.1111/1471-0528.16276

**Published:** 2020-05-26

**Authors:** Y Wu, C Liu, L Dong, C Zhang, Y Chen, J Liu, C Zhang, C Duan, H Zhang, BW Mol, C‐L Dennis, T Yin, J Yang, H Huang

**Affiliations:** ^1^ The International Peace Maternity and Child Health Hospital School of Medicine Shanghai Jiao Tong University Shanghai China; ^2^ Department of Radiology First Affiliated Hospital to Army Medical University Chongqing China; ^3^ Renmin Hospital of Wuhan University Wuchang, Wuhan China; ^4^ Wuhan Children’s Hospital (Wuhan Maternal and Child Healthcare Hospital) Tongji Medical College Huazhong University of Science & Technology Wuhan China; ^5^ Department of Obstetrics and Gynaecology Monash University Clayton Victoria Australia; ^6^ Bloomberg Faculty of Nursing University of Toronto Toronto Ontario Canada

**Keywords:** Breast milk, coronavirus disease 2019, vaginal secretions

## Abstract

**Objective:**

To assess whether vaginal secretions and breast milk of women with coronavirus disease 2019 (COVID‐19) contain severe acute respiratory syndrome coronavirus 2 (SARS‐CoV‐2).

**Design:**

Single centre cohort study.

**Setting:**

Renmin Hospital of Wuhan University, Wuhan, Hubei province, China.

**Population:**

We studied 13 SARS‐CoV‐2‐infected pregnant women diagnosed between 31 January and 9 March 2020.

**Methods:**

We collected clinical data, vaginal secretions, stool specimens and breast milk from SARS‐CoV‐2‐infected women during different stages of pregnancy and collected neonatal throat and anal swabs.

**Main outcomes and measures:**

We assessed viral presence in different biosamples.

**Results:**

Of the 13 women with COVID‐19, five were in their first trimester, three in their second trimester and five in their third trimester. Of the five women in their third trimester who gave birth, all delivered live newborns. Among these five deliveries, the primary adverse perinatal outcomes included premature delivery (*n* = 2) and neonatal pneumonia (*n* = 2). One of nine stool samples was positive; all 13 vaginal secretion samples, and five throat swabs and four anal swabs collected from neonates, were negative for the novel coronavirus. However, one of three samples of breast milk was positive by viral nucleic acid testing.

**Conclusions:**

In this case series of 13 pregnant women with COVID‐19, we observed negative viral test results in vaginal secretion specimens, suggesting that a vaginal delivery may be a safe delivery option. However, additional research is urgently needed to examine breast milk and the potential risk for viral contamination.

**Tweetable abstract:**

New evidence for the safety of vaginal delivery and breastfeeding in pregnant women infected with SARS‐CoV‐2, positive viral result in a breast‐milk sample.

## Abstract 2

New evidence for the safety of vaginal delivery and breastfeeding in pregnant women infected with SARS‐CoV‐2, positive viral result in a breast‐milk sample.

## Introduction

In December of 2019, Wuhan, in Hubei Province, China, experienced an outbreak of pneumonia caused by the severe acute respiratory syndrome coronavirus 2 (SARS‐CoV‐2). On 11 March the World Health Organization declared that the spread of coronavirus disease 2019 (COVID‐19) was a pandemic, which is now disrupting health and public life around the world.^1^

SARS‐CoV‐2 shares 79% nucleotide sequence identity with SARS‐CoV and 50% with Middle‐East respiratory syndrome coronavirus (MERS‐CoV).^2^ Previous studies have suggested that SARS‐CoV infection during pregnancy may carry severe complications including maternal death, spontaneous miscarriage, preterm delivery and intrauterine growth restriction;^3^ and MERS has been associated with intrauterine fetal demise and stillbirth.^4^, ^5^

Human transmission is through droplets and direct contact, though recent investigations have demonstrated that SARS‐CoV‐2 is also detectable in stool and blood.^6^ No evidence of vertical transmission was demonstrated in nine pregnant women, and amniotic fluid, cord blood and breast milk samples of six women were all negative.^7^ However, one newborn was diagnosed with SARS‐CoV‐2 infection within 36 hours of delivery, which raised the concern of vertical transmission.^8^ Viral analysis of the vaginal mucosa or secretions has not been reported, so there may be a clinical tendency to promote caesarean delivery to avoid intrapartum transmission.^7^ Furthermore, data on breastfeeding are scarce and no one has examined the effect of COVID‐19 in the first and second trimesters of pregnancy. In view of these clinical gaps, we performed the retrospective study.

## Methods

All pregnant women with SARS‐CoV‐2 admitted to Renmin Hospital of Wuhan University in China between 31 January and 9 March 2020, and their babies admitted to Wuhan Children’s Hospital (Wuhan Maternal and Child Healthcare Hospital), Tongji Medical College, Huazhong University of Science & Technology, were included. The diagnostic criteria for COVID‐19 were based on the Diagnosis and Treatment Protocol for the 2019 Novel Coronavirus Pneumonia published by the National Health Commission of China.^9^ All patients were diagnosed through real‐time reverse transcription–polymerase chain reaction assays of throat swab samples. This study was approved by the Medical Ethical Committee of Renmin Hospital of Wuhan University (No. WDRY2020‐K097), Wuhan Children’s Hospital (Wuhan Maternal and Child Healthcare Hospital), Tongji Medical College, Huazhong University of Science &T echnology (No. WHCH 2020014), and the International Peace Maternity and Child Health Hospital, School of Medicine, Shanghai Jiao Tong University (No. GKLW2020‐05).

We extracted demographic information, clinical course, laboratory indices and imaging results of infected pregnant women from the medical records, maternal throat swabs were collected upon admission. Samples of vaginal secretions from nine women were collected during pregnancy and samples from four women were collected after delivery. Neonatal throat and anal swabs were collected on the 1st and 3rd days after birth. Breast milk samples from three women were collected on the 1st, 6th and 27th days after delivery and stool specimens were taken from nine women between 5 and 8 March. The details of sample collection are shown in the Supplementary material (Tables S1 and S2). We collected throat swabs, anal swabs, vaginal secretions and stool specimens using sterile polyester swabs. After collection, samples were placed in sterile tubes and transported to the laboratory at 4°C. For breast‐milk collection, iodine was used to disinfect the patient’s breast. Breast milk was expressed into a sterile container. Maternal samples were processed at the Laboratory of Renmin Hospital of Wuhan University, and neonatal samples were processed at the Laboratory of Wuhan Children’s Hospital. Both laboratories met the World Health Organization standard.^10^

## Results

We reviewed the medical records of 13 pregnant women with SARS‐CoV‐2 infection; where five were infected in the first trimester, three in the second trimester and five in the third trimester. One of the infants included here has been previously reported.^11^ All women had a history of viral exposure in the central epidemic area in and around Wuhan. The women were between 26 and 40 years of age, with parity ranging from 0 to 2; hospital admission for infection occurred between 5 and 38 weeks of gestation. All women were diagnosed as having mild COVID‐19 without severe complications, and none was admitted to the intensive care unit. All women were provided with oxygen support, antiviral therapy assisted by antibacterial treatment was administered to eight women, and corticosteroid treatment was given to three women. All 13 women are currently virus free (see Supplementary material, Table S3).

In terms of clinical manifestations, fever was the most common symptom (*n* = 8); followed by cough (*n* = 5), dyspnoea (*n* = 1), myalgia (*n* = 1) and diarrhoea (*n* = 1). Three women demonstrated an increased leucocyte count, two women had lymphopenia and five women had elevated C‐reactive protein levels. Impaired liver function was observed in three women (with elevated levels of alanine aminotransferase), and two women had increased aspartate aminotransferase levels. Eight women manifested patchy ground‐glass opacities or consolidation shadows, with peripherally distributed computed tomography imaging features.

### Perinatal outcomes

In the five women in the first trimester of pregnancy, one (patient 12) had a biochemical pregnancy with serum human chorionic gonadotrophin concentrations dropping from 25.9 IU/l to <2 IU/l. The other four women (patients 7, 9, 10 and 13) had ongoing pregnancies. All five women in their third trimester (patients 1–5) delivered, with only one (patient 5) undergoing vaginal birth. While patient 1 experienced dyspnoea – with fetal heart rate monitoring that suggested fetal distress – the other three women exhibited no clear medical indication for a caesarean section. Lack of knowledge regarding COVID‐19 and concerns about potential mother‐to‐child transmission as a ‘social factor’ became an indication for caesarean section in these last three pregnant women (Table 1). The gestational age at delivery ranged from 35^+5^ to 38^+4^ weeks. Two neonates (babies of patients 1 and 2) were born prematurely (at 35^+5^ and 35^+6^ weeks), with birthweights of 2830 g and 2300 g, respectively. The pregnancy of patient 1 was ended prematurely because of fetal distress, and patient 2 underwent a spontaneous preterm birth. The neonate of patient 5 was large for gestational age (at 3910 g) (see Supplementary material, Table S4). Two of the five women had increased leucocyte counts, and three of the five women continued to show elevated C‐reactive protein levels after delivery (Table 1).

**Table 1:** Childbirth characteristics

	Patient 1	Patient 2	Patient 3	Patient 4	Patient 5	*n* (%)
Gestational age at delivery	35^+6^ weeks	35^+5^ weeks	38^+4^ weeks	37^+6^ weeks	38^+2^ weeks	–
Onset to delivery (days)	11	0	NK	25	0	–
Method of delivery	Caesarean section	Caesarean section	Caesarean section	Caesarean section	Natural delivery	–
Indication for caesarean section	Fetal distress	Social factor*	Social factor	Social factor	NA	–
**Laboratory characteristics (after delivery)**						
White blood cell count (×10^9^ cells/l)	7.06	8.41	8.25	11.18	29.53	–
Low or normal leucocyte count (<9.5 × 10^9^ cells/l)	Yes	Yes	Yes	No	No	3 (60%)
Lymphocyte count (×10^9^ cells/l)	1.72	1.07	2.06	1.28	0.58	–
Lymphopenia (<10^9^ cells/l)	No	No	No	No	Yes	1 (20%)
C‐reactive protein concentration (mg/l)	29.1	64.0	8.7	87.6	ND	–
Elevated C‐reactive protein (>10 mg/l)	Yes	Yes	No	Yes	NA	–
Intraoperative blood loss (ml)	200 ml	200 ml	200 ml	300 ml	NA	–
2 hours postpartum bleeding (ml)	300 ml	300 ml	300 ml	300 ml	100 ml	–
Detection of SARS‐CoV‐2 in breast milk	Negative	ND	ND	Negative	Positive	–

NA, not applicable; ND, not done; NK, not known.

* Social factor: these women had no medical indication for caesarean section, and the patient’s choice of caesarean section was attributed to social reasons in the medical records.

All five women delivered a live newborn; the infant of patient 3 had a 1‐min Apgar score of 7 and 5‐min Apgar score of 9, but the other four infants had normal Apgar scores. Two neonates (babies of patient 1 and patient 2) were born prematurely and diagnosed with neonatal pneumonia by chest X‐ray (see Supplementary material, Figure S1). SARS‐CoV‐2 nucleic acid tests of neonatal throat and anal swabs were all negative on the 1st and 3rd days after birth.

### Viral nucleic acid test results of vaginal secretions and breast milk

**Figure 1:**
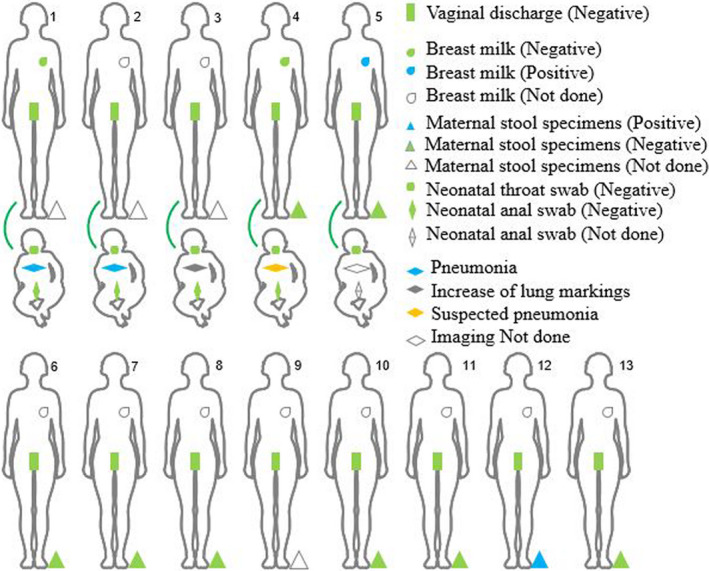
Summary of virus detection in pregnant women with COVID‐19. Samples of vaginal secretions, breast milk and stool were collected from 13 pregnant women with SARS‐CoV‐2 infection. Four of the pregnant women in their third trimester (Patients 1 to 4) had caesarean sections, one woman (patient 5) had a vaginal birth. The neonatal throat and anal swabs were collected on the 1st and 3rd days after birth to detect the COVID‐19 virus. Four neonates received chest X‐rays or computed tomography imaging on the 2nd day after birth.

All 13 vaginal secretions samples were negative for viral nucleic acids and only one stool sample was positive (patient 12) (see Supplementary material, Table S3); the viral nucleic acid tests of throat swabs of ten women were positive on the day of vaginal secretion collection (see Supplementary material, Table S1). We assessed breast milk in three women; the breast‐milk sample of one woman (patient 5), collected on the 1st day after delivery, was positive using the real‐time reverse transcription–polymerase chain reaction test for the open reading frame 1ab of SARS‐CoV‐2 (see Supplementary material, Table S3), but subsequent re‐examination on the 3rd day after delivery was negative. The other breast‐milk samples, collected on the 6th and 27th days after delivery, were negative for the virus (see Supplementary material, Table S1). The viral‐detection results for all participants are summarised in Figure 1.

Data from neonate 4, born in Renmin Hospital, suggested no diagnosis of SARS‐CoV‐2 infection; this newborn had four negative swabs, no clinical symptoms or signs and received no treatment. However, the newborn was subsequently found to have IgM and IgG seroconversion. This newborn has been previously reported elsewhere.^11^

## Discussion

### Main findings

In this study, the clinical characteristics of the pregnant women were mild compared with the general Chinese population, and none of them progressed to severe pneumonia.^12^ SARS‐CoV‐2 was not found in the 13 vaginal swabs, and none of the five newborns was infected, although one of the three breast‐milk samples was positive for the virus.

### Strengths and limitations

Our study is the first to report on 13 women at various stages of pregnancy with laboratory‐confirmed SARS‐CoV‐2 infection, and on the ensuing safety of a vaginal birth and breastfeeding. Our study has some limitations. First, only 13 pregnant women with SARS‐CoV‐2 infection and five newborns were included in our study; therefore, additional studies with larger sample sizes are required to confirm our preliminary results. Furthermore, there were no pregnant women with severe symptoms requiring intensive care in our study, limiting the clinical implications. Also, all women in our study were from the Renmin Hospital of Wuhan University, so it is not known if our results are applicable to other medical centres. Given the widespread nature of this global public health emergency, there may be different clinical characteristics in different COVID‐19‐epidemic areas due to differences in race, culture, policies and medical conditions. Further studies involving additional populations and multiple centres are required to confirm our results. Finally, this was a retrospective study, which limited our ability to examine the long‐term health effects on infants born to mothers with COVID‐19.

### Interpretation

Vertical transmission is a key concern for pregnant women with SARS‐CoV‐2 infection. None of the newborns delivered in our study was infected, a result consistent with previous reports (e.g. negative tests for the novel coronavirus nucleic acid in pharyngeal swab samples from 19 neonates born to mothers with COVID‐19 pneumonia, and for three placental samples^13^, ^14^). Two newborns with neonatal coronavirus pneumonia were confirmed in Wuhan^8^, ^15^ as of 5 February 2020; one of them was diagnosed with SARS‐CoV‐2 infection 36 hours after birth. Expert clinical opinion suggests that this SARS‐CoV‐2 infection was the result of close contact with an infected individual rather than to vertical transmission. In our study, one woman gave birth vaginally and the newborn’s viral nucleic acid test was negative. However, it is unclear whether intrauterine transmission of SARS‐CoV‐2 occurs, and additional research is therefore warranted.

Intrapartum transmission is another concern that may affect mode of delivery. Many types of viruses – including herpes simplex virus, human papillomavirus and HIV – can be spread to neonates by intrapartum transmission.^16^, ^17^, ^18^ An important mechanism for viral intrapartum transmission is through swallowing of infected vaginal secretions during vaginal delivery. In this study, three of four caesarean sections were performed without any medical indications in an attempt to avoid potential intrapartum transmission. Importantly, we collected vaginal secretions from the 13 women for viral nucleic acid testing to clarify whether SARS‐CoV‐2 could be transmitted via vaginal delivery; and our test results were negative in amniotic fluids and placentae from nine women, which should reassure women who wish to deliver vaginally and can do so.^7^, ^14^ The results of our study support the recommendation that all infected pregnant women with COVID‐19 who do not have a medical indication for a caesarean section should have a vaginal delivery.^19^

Some pathogens – such as herpes simplex virus and *Chlamydia trachomatis* – can travel along the genital tract and spread to the uterus, causing adverse neonatal outcomes such as stillbirth and premature birth.^20^, ^21^, ^22^ Our negative results of vaginal secretions from 13 women, however, suggest that ascending infection in COVID‐19 was low. An important mechanism underlying the entry of SARS‐CoV‐2 into human cells has been demonstrated through the interaction between viral S‐protein and angiotensin‐converting enzyme 2 (ACE2) in the host.^2^, ^23^ Therefore, the distribution of ACE2 expression may be closely associated with target‐organ damage and viral transmission. Although RNA and protein expression levels for ACE2 are very low in the vagina, uterus and cervix, the ACE2 receptor is expressed in the digestive tract and oral mucosa.^24^, ^25^ This may explain why SARS‐CoV‐2 can be found in human saliva and faeces rather than in vaginal secretions. While we could not find evidence for intrapartum transmission of SARS‐CoV‐2, a previous case report from our series indicated intrauterine seroconversion of a neonate. This neonate showed no signs of sickness after birth. Seroconversion was found only in this pregnancy.^11^

Advice regarding breastfeeding is clearly a matter of concern. Previous studies have found that HIV and hepatitis B virus appear in breast milk and cause mother‐to‐infant transmission;^26^, ^27^ and that mothers with COVID‐19 can transmit the virus through respiratory droplets or skin contact during breastfeeding. In contrast to a study that did not find SARS‐CoV‐2 in the breast milk of women with COVID‐19,^7^ we found a positive viral nucleic acid test in one breast‐milk sample. Although we retested the breast milk from this infected women 2 days later and found it to be negative, the possibility of viral transmission through breast milk cannot be excluded. Until large studies have demonstrated the safety of breast milk, our advice is against the use of breastfeeding even through breast expression; mothers with COVID‐19 should not breastfeed until after full recovery, when breast milk tests negative for the virus.

Our study confirms that pregnant women with SARS‐CoV‐2 are not at high risk of severe illness and adverse pregnancy outcomes, which is in accordance with previous studies.^7^, ^28^ Previous studies have shown that pregnant women with SARS are prone to adverse maternal and neonatal complications, including miscarriage, preterm birth, intrauterine growth restriction, use of intubation and admission to the intensive care unit, and have a mortality rate of 25%.^29^ In the present study, pregnant women with COVID‐19, in contradistinction, exhibited far fewer adverse maternal and neonatal complications and outcomes. One reason for the discrepancy may be that SARS‐CoV has a significantly greater virulence than SARS‐CoV‐2, causing SARS‐CoV‐infected patients to generally manifest more severe signs and symptoms than SARS‐CoV‐2‐infected patients; and pregnant women have a poor prognosis when suffering from severe pneumonia.

We also observed one pregnancy failure, raising concerns that SARS‐CoV‐2 infection may induce early pregnancy loss. The aetiology of a biochemical pregnancy is diverse and may be the result of thrombophilic disorders, chromosomal anomalies, various endocrine disturbances, immune dysfunction or disrupted endometrium.^30^ In addition, infection is a major cause of miscarriage; overall, 15% of early miscarriages and 66% of late miscarriages are related to infections.^31^ The association between a systemic infection such as malaria, dengue fever, brucellosis, cytomegalovirus, HIV, influenza or bacterial vaginosis, and an increased risk of miscarriage has been well documented.^31^ The mechanisms of termination of pregnancy caused by the influenza virus, SARS CoV or MERS CoV comprise maternal respiratory difficulties and insufficient delivery of oxygen to the embryo, and are not due to direct viral infection of the fetus.^2^, ^3^, ^32^ The woman in our study who experienced the biochemical pregnancy did not experience dyspnoea, so fetal oxygenation was probably sufficient. It is unclear whether the biochemical pregnancy was associated with maternal infection, and therefore additional research is warranted.

## Conclusion

In summary, in our study we found no evidence for vertical or intrapartum transmission of the novel coronavirus, with negative test results for all of our vaginal samples. One infected women gave birth vaginally to a healthy newborn, providing additional evidence that a vaginal delivery among infected women is a safe option. However, SARS‐CoV‐2 could potentially be transmitted through breast milk, and therefore further research is urgently needed. The impact of SARS‐CoV‐2 infection on embryo health during the first trimester of pregnancy also requires further investigation. With the global outbreak of COVID‐19, we believe that our findings provide health professionals with important clinical information when treating infected pregnant women and providing perinatal care.

### Disclosure of interests

BWM is supported by an NHMRC Investigator grant (GNT1176437). BWM reports consultancies for ObsEva, Merck KGaA, iGenomix and Guerbet, with no potential conflicts of interest. The other 13 authors have no conflicts of interest. Completed disclosure of interests forms are available to view online as supporting information.

### Contribution to authorship

HH, JY and TY contributed to concept and design. YW, CJZ, CZ, CD, HZ, BWM and CLD drafted the manuscript. CZ and CD performed the statistical analyses and CL, LD, YC and JL collected the data.

### Details of ethics approval

This study was approved by the Medical Ethical Committee of Renmin Hospital of Wuhan University (No. WDRY2020‐K097; date: March 2020), Wuhan Children’s Hospital (Wuhan Maternal and Child Healthcare Hospital), Tongji Medical College, Huazhong University of Science & Technology (No. WHCH 2020014; date 7 March 2020) and the International Peace Maternity and Child Health Hospital, School of Medicine, Shanghai Jiao Tong University (No. GKLW2020‐05; date 15 March 2020).

### Funding/support

This work was supported by the National Key Research and Development Programme of China (2018YFC1002804, 2016YFC1000203). The funders had no role in the design or conduct of this study; collection, management, analysis, or interpretation of the data; preparation, review, or approval of the manuscript; or decision to submit this manuscript for publication.

### Acknowledgements

We wish to thank the women, researchers and clinical staff who provided significant contributions to this study.

## Supporting information


**Figure S1.** Imaging examination of the four delivered women and their neonates.

**Table S1. **Data of maternal sample collection.
**Table S2. **Data of neonatal sample collection.
**Table S3. **Samples of clinical and laboratory characteristics.
**Table S4. **Neonatal outcomes.

Supplementary Material

Supplementary Material

Supplementary Material

Supplementary Material

Supplementary Material

Supplementary Material

Supplementary Material

Supplementary Material

Supplementary Material

Supplementary Material

Supplementary Material

Supplementary Material

Supplementary Material

Supplementary Material
