# Supplementary material for: Coronavirus disease 2019 among pregnant Chinese women: case series data on the safety of vaginal birth and breastfeeding
Source: BJOG. 2020 May 26;127(9):1109–15. doi: 10.1111/1471-0528.16276 (PMC7383704; doi:10.1111/1471-0528.16276)
Supplement: Supplementary file 1 — Figure S1. Imaging examination of the four delivered women and their neonates. [file BJO-127-1109-s001.pdf]

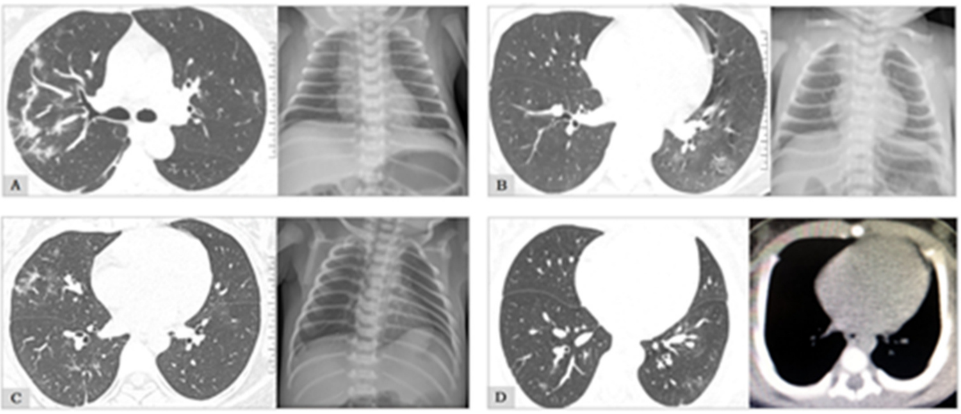

Figure legend: (A) Patient 1 (left, chest CT), bilateral multiple ground-glass densities; neonate of patient 1 (right, chest radiography), pneumonia; (B) patient 2 (left, chest CT), bilateral multiple ground-glass densities; neonate of patient 2 (right, chest radiography), pneumonia; (C) patient 3 (left, chest CT), bilateral ground-glass opacities with diffuse lesions; neonate of patient 3 (right, chest radiography), bilateral increased bronchovascular shadows; (D) patient 4 (left, chest CT), bilateral multiple ground-glass opacities; neonate of patient 4 (right, chest CT), suspected pneumonia.

Figure S1. Imagining examination of the 4 delivered women and their neonates.
