# Supplementary material for: Coronavirus disease 2019 among pregnant Chinese women: case series data on the safety of vaginal birth and breastfeeding
Source: BJOG. 2020 May 26;127(9):1109–15. doi: 10.1111/1471-0528.16276 (PMC7383704; doi:10.1111/1471-0528.16276)
Supplement: Supplementary file 2 — Table S1. Data of maternal sample collection. Table S2. Data of neonatal sample collection. Table S3. Samples of clinical and laboratory characteristics. Table S4. Neonatal outcomes. [file BJO-127-1109-s002.pdf]

Table S1. Dates of maternal sample collection

	Patient 1	Patient 2	Patient 3	Patient 4	Patient 5	Patient 6	Patient 7	Patient 8	Patient 9	Patient 10	Patient 11	Patient 12	Patient 13
Date of admission	Feb 1	Feb 3	Feb 14	Feb 2	Mar 1	Mar 4	Feb 9	Feb 9	Feb 5	Feb 23	Feb 15	Feb 13	Feb 25
Date of delivery	Feb 2	Feb 3	Feb 14	Feb 22	Mar 1	NP	NP	NP	NP	NP	NP	NP	NP
Date of laboratory examination (before delivery)	Feb 1	Feb 3	Feb 14	Feb 2	Mar 1	Mar 4	Feb 9	Feb 9	Feb 5	Feb 23	Feb 15	Feb 13	Feb 25
Results of the patient's throat swab for viral nucleic acid on the day of laboratory examination (before delivery)[c]	Positive	Positive	Positive	Positive	Positive	Positive	Positive	Positive	Positive	Positive	Positive	Positive	Positive
Date of laboratory examination (after delivery)	Feb 5	Feb 7	Feb 22	Feb 25	Mar 1	NP	NP	NP	NP	NP	NP	NP	NP
Results of the patient's throat swab for viral nucleic acid on the day of laboratory examination (after delivery)[c]	Positive	Positive	Negative	Positive	Positive	NP	NP	NP	NP	NP	NP	NP	NP
Date of vaginal secretions collection	Feb 19	Feb 19	Feb 19	Feb 28	Mar 6	Mar 6	Mar 8	Feb 19	Mar 8	Feb 26	Mar 2	Mar 4	Feb 28
Results of the patient's throat swab for viral nucleic acid on the day of vaginal secretions collection[c]	Positive	Positive	NK	Negative	Positive	Positive	Positive	Positive	Positive	Positive	Positive	Positive	Positive

Date of breast milk collection	Feb 29	ND	ND	Feb 28	Mar 2	ND	ND	ND	ND	ND	ND	ND	ND
Results of the patient's throat swab for viral nucleic acid on the day of breast milk collection[c]	Negative	NP	NP	Positive	NK	NP	NP	NP	NP	NP	NP	NP	NP
Date of stool specimen collection	ND	ND	ND	Mar 5	Mar 5	Mar 8	Mar 8	Mar 5	ND	Mar 8	Mar 8	Mar 8	Mar 8
Results of the patient's throat swab for viral nuclei acid on the day of stool specimen collection[c]	NP	NP	NP	NK	Negative	Positive	NK	NK	NP	Negative	Negative	Positive	Positive

NK=Not known; ND=Not Done; NA=Not applicable

[c]Normally, testing of patients' throat swabs for viral nucleic acid and sample collection were not performed on the same day. If the viral nucleic acid testing of throat swabs was positive after the patient sample collection date, we considered the test to be "positive" on the day of sample collection. If the most recent viral nucleic acid testing before and after the day of sample collection was negative, we considered the test on the day of sample collection to be "negative". If the latest viral nucleic acid test of throat swabs was positive before the day of sample collection, and the subsequent throat swab test was negative, we did not know (NK) the throat swab testing result on the day of sample collection.

Table S2. Date of neonatal sample collection

	Patient 1	Patient 2	Patient 3	Patient 4	Patient 5
Date of birth	Feb 2	Feb 3	Feb 14	Feb 22	Mar 1
Results from the maternal throat swab for viral nucleic acid on the day of delivery*	Positive	Positive	Positive	Positive	Positive
Date of neonatal throat swab collection (the first time)	Feb 3	Feb 4	Feb15	Feb 23	Mar 2
Date of neonatal anal swab collection (the first time)	Feb 3	Feb 4	Feb15	Feb 23	Mar 2
Date of neonatal throat swab collection (the second time)	Feb 5	Feb 6	Feb 17	Feb 25	Mar 4
Date of neonatal anal swab collection (the second time)	Feb 5	Feb 6	Feb 17	Feb 25	Mar 4

Table S3. Samples of clinical and laboratory characteristics

	Patient 1	Patient 2	Patient 3	Patient 4	Patient 5	Patient 6	Patient 7	Patient 8	Patient 9	Patient 10	Patient 11	Patient 12	Patient 13	n (%)
Clinical characteristics														
Date of onset	Jan 22	Feb 3	NK	Jan 28	Mar 1	Mar 2	Jan 26	Feb 4	Jan 25	Feb 20	Jan 31	Feb 5	Feb 18	-
Date of admission	Feb 1	Feb 3	Feb 14	Feb 2	Mar 1	Mar 4	Feb 9	Feb9	Feb 5	Feb 23	Feb 15	Feb 13	Feb 25	-
Onset to admission (days)	10	0	NK	5	0	2	14	5	11	3	15	8	7	-
Age (years)	29	26	29	28	27	31	40	36	28	27	29	32	35	-
Height (cm)	163	165	163	155	162	169	155	155	172	172	167	155	156	-
Weight (Kg)	70	48	60	55	51	54	49	53	67.5	65	65	57	55	-
BMI	26.3	17.6	22.6	22.9	19.4	18.9	20.4	22.1	22.8	22.0	19.7	23.7	22.6	-
Gravidity	2	1	2	1	2	3	4	2	2	1	1	1	2	-
Parity	1	1	2	1	1	1	2	1	0	0	0	1	1	-

Miscarriage history	Yes	No	No	No	Yes	Yes	Yes	No	Yes	No	No	No	No	5 (38%)
Health care worker	No	No	No	No	No	No	No	No	No	No	No	No	No	0
Gestational age upon admission	35^{+5} weeks	35^{+5} weeks	38^{+4} weeks	35 weeks	38^{+2} weeks	32^{+4} weeks	8^{+5} weeks	16^{+1} weeks	6^{+3} weeks	11^{+5} weeks	18 weeks	5^{+1} weeks	9^{+3} weeks	-
Gestational weeks at diagnosis of COVID-19	35^{+5} weeks	35^{+6} weeks	38^{+4} weeks	35^{+4} weeks	38^{+2} weeks	32^{+2} weeks	8^{+2} weeks	15^{+6} weeks	5^{+3} weeks	11^{+2} weeks	17 weeks	4^{+2} weeks	9 weeks	-
Epidemiology history	Yes (Exposure to relevant environment)[a]	Yes (Exposure to relevant environment)	Yes (Exposure to relevant environment)	Yes (Contact with infected person)	Yes (Exposure to relevant environment)	Yes (Exposure to relevant environment)	Yes (Exposure to relevant environment)	Yes (Exposure to relevant environment)	Yes (Exposure to relevant environment)	Yes (Contact with infected person)	Yes (Exposure to relevant environment)	Yes (Exposure to relevant environment)	Yes (Exposure to relevant environment)	13 (100%)
Complications	None	None	None	Thrombocytopenia	PROM	None	None	None	None	None	Chronic nephritis	None	None	3 (23%)

Patient condition	Mild	Mild	Mild	Mild	Mild	Mild	Mild	Mild	Mild	Mild	Mild	Mild	Mild	-
**Signs and symptoms**														
Fever	Yes	No	No	Yes	No	No	Yes	Yes	Yes	No	Yes	Yes	Yes	8 (62%)
Post-partum fever	No	No	No	No	No	No	No	No	No	No	No	No	No	0
Myalgia	No	No	No	No	No	No	No	No	No	No	No	Yes	No	1 (8%)
Malaise	No	No	No	No	No	No	No	No	No	No	No	No	No	0
Rigor	No	No	No	No	No	No	No	No	No	No	No	No	No	0
Cough	Yes	No	No	No	No	No	Yes	Yes	Yes	No	No	No	Yes	5(38%)
Dyspnea	Yes	No	No	No	No	No	No	No	No	No	No	No	No	1 (8%)
Sore throat	No	No	No	No	No	No	No	No	No	No	No	No	No	0
Diarrhea	Yes	No	No	No	No	No	No	No	No	No	No	No	No	1 (8%)
Chest pain	No	No	No	No	No	No	No	No	No	No	No	No	No	0
**Laboratory characteristics**														

**(Before delivery)**														
White blood cell count ($\times 10^9$ cells/L)	4.28	8.59	17.37	8.03	12.09	6.27	4.98	4.58	3.95	7.43	11.00	5.59	10.20	-
Low or normal leukocyte count ($<9.5\times 10^9$ cells/L)	Yes	Yes	No	Yes	No	Yes	Yes	Yes	Yes	Yes	Yes	Yes	No	10 (77%)
Lymphocyte count ($\times 10^9$ cells/L)	1.01	0.89	1.61	1.08	1,06	1.07	1.51	0.88	1.41	1.93	2.11	1.09	1.88	-
Lymphopenia ($<10^9$ cells/L)	No	Yes	No	No	No	No	No	Yes	No	No	No	No	No	2 (15%)
C-reactive protein concentration (mg/L)	53.2	38.5	<5.0	57.0	<5.0	<5.0	<5.0	66.1	<5.0	<5.0	<5.0	<5.0	83.2	-
Elevated C-reactive protein (>10 mg/L)	Yes	Yes	No	Yes	No	No	No	Yes	No	No	No	No	Yes	5 (38%)
Elevated ALT (>45 U/L) or	No	No	No	Yes	No	No	No	No	Yes	No	No	No	Yes	3 (23%)

AST (>35 U/L)														
ALT (U/L)	13	12	15	40	6	9	29	11	115	20	9	31	54	-
AST (U/L)	26	23	19	38	13	14	24	23	80	13	15	24	32	-
Diagnostic method	RT-PCR	RT-PCR	RT-PCR	RT-PCR	RT-PCR	RT-PCR	RT-PCR	RT-PCR	RT-PCR	RT-PCR	RT-PCR	RT-PCR	RT-PCR	-
Typical signs of viral infection (CT evidence of pneumonia)	Yes	Yes	Yes	Yes	No	ND	Yes	Yes	ND	Yes	ND	Yes	No	-
Detection of SARS-CoV-2 in vaginal secretions	Negative	Negative	Negative	Negative	Negative	Negative	Negative	Negative	Negative	Negative	Negative	Negative	Negative	0
Detection of SARS-CoV-2 in maternal stool specimens	ND	ND	ND	Negative	Negative	Negative	Negative	Negative	ND	Negative	Negative	Positive	Negative	-
**Treatment**														
Oxygen support (nasal	Yes	Yes	Yes	Yes	Yes	Yes	Yes	Yes	Yes	Yes	Yes	Yes	Yes	13

cannula)														(100%)
Antiviral therapy	Oseltamivir, arbidole, ribavirin, hydroxychloroquine	Oseltamivir, arbidole, ribavirin, hydroxychloroquine	Arbidole	Oseltamivir, arbidole, ribavirin	Arbidole	Arbidole	Arbidole	Arbidole	Oseltamivir, arbidole	Arbidole	Arbidole	Oseltamivir, arbidole	Oseltamivir, arbidole	13 (100%)
Antibiotic therapy	No	No	No	No	Cefoperazone and tazobactam	No	Cefoperazone and tazobactam, moxifloxacin	Azithromycin, cefoperazone and tazobactam, cefaclor	Cefoperazone and tazobactam	Cefathiamidine	Azithromycin	Cefoperazone and tazobactam	Cefoperazone and tazobactam, azithromycin	8 (62%)

Use of corticosteroid	Methylprednisolone	No	No	Methylprednisolone	No	No	Methylprednisolone	No	No	No	No	No	No	3 (23%)
Pregnancy outcomes	Live birth	Live birth	Live birth	Live birth	Live birth	In pregnancy	In pregnancy	In pregnancy	In pregnancy	In pregnancy	In pregnancy	Chemical pregnancy	In pregnancy	-
Current situation	Cured	Cured	Cured	Cured	Cured	Cured	Cured	Cured	Cured	Cured	Cured	Cured	Cured	-

NK=Not known; ND=Not Done; ALT=alanine transaminase; AST=aspartate transaminase; BMI=Body mass index; CT=Computed tomography; PROM=Premature rupture of membrane. [a]Exposure to relevant environment: Due to the outbreak in Wuhan, and these women live in Wuhan. Therefore, although there was no clear history of contact with COVID-19 patients, they were still considered to have a risk of infection.

Table S4. Neonatal outcomes

	Patient 1	Patient 2	Patient 3	Patient 4	Patient 5	n (%)
Birth weight (g)	2830	2300	2650	3120	3910	-
Low birth weight (<2500 g)	No	Yes	No	No	No	1 (20%)
Premature delivery	Yes	Yes	No	No	No	2 (40%)
Small for gestational age (<10th %ile)	No	No	No	No	No	0
Appropriate for gestational age	Yes	Yes	Yes	Yes	No	4 (80%)
Large for gestational age (>90th %ile）	No	No	No	No	Yes	1 (20%)
Apgar score (1 min, 5 min)	9, 10	9, 10	7, 9	9, 10	9, 10	-
Neonatal asphyxia	No	No	Yes	No	No	1 (20%)
Neonatal death	No	No	No	No	No	0
Fetal death or stillbirth	No	No	No	No	No	0
Detection of SARS-CoV-2 in neonate	Negative	Negative	Negative	Negative	Negative	0

(throat swab, the first day after delivery)						
Detection of SARS-CoV-2 in neonate (anal swab, the first day after delivery)	Negative	Negative	Negative	Negative	ND	-
Detection of SARS-CoV-2 in neonate (throat swab, the third day after delivery)	Negative	Negative	Negative	Negative	ND	-
Detection of SARS-CoV-2 in neonate (anal swab, the third day after delivery)	Negative	Negative	Negative	Negative	ND	-
Imaging results (days after birth)	Pneumoni a	Pneumoni a	Increase of lung markings	Suspected pneumoni a	ND	-

ND=Not done.
